# A Temporal Boosted YOLO-Based Model for Birds Detection around Wind Farms

**DOI:** 10.3390/jimaging7110227

**Published:** 2021-10-27

**Authors:** Hiba Alqaysi, Igor Fedorov, Faisal Z. Qureshi, Mattias O’Nils

**Affiliations:** 1 Department of Electronics Design, Mid Sweden University, Holmgatan 10, 85170 Sundsvall, Sweden; mattias.onils@miun.se; 2 Department of Science and Technology, Linköping University, 58183 Linköping, Sweden; igor.fedorov@liu.se; 3 Faculty of Science, University of Ontario Institute of Technology, Oshawa, ON L1G 0C5, Canada; faisal.qureshi@ontariotechu.net

**Keywords:** bird detection, wind farms monitoring, sky surveillance, background subtraction, YOLOv4

## Abstract

Object detection for sky surveillance is a challenging problem due to having small objects in a large volume and a constantly changing background which requires high resolution frames. For example, detecting flying birds in wind farms to prevent their collision with the wind turbines. This paper proposes a YOLOv4-based ensemble model for bird detection in grayscale videos captured around wind turbines in wind farms. In order to tackle this problem, we introduce two datasets—(1) Klim and (2) Skagen—collected at two locations in Denmark. We use Klim training set to train three increasingly capable YOLOv4 based models. Model 1 uses YOLOv4 trained on the Klim dataset, Model 2 introduces tiling to improve small bird detection, and the last model uses tiling and temporal stacking and achieves the best mAP values on both Klim and Skagen datasets. We used this model to set up an ensemble detector, which further improves mAP values on both datasets. The three models achieve testing mAP values of 82%, 88%, and 90% on the Klim dataset. mAP values for Model 1 and Model 3 on the Skagen dataset are 60% and 92%. Improving object detection accuracy could mitigate birds’ mortality rate by choosing the locations for such establishment and the turbines location. It can also be used to improve the collision avoidance systems used in wind energy facilities.

## 1. Introduction

Wind power is rapidly emerging as a viable green energy alternative to fossil fuel. Wind farms installations that are needed to generate electricity from wind power can interfere with bird migrations—for example, birds can perish upon collision with wind turbines. It is estimated that up to 500,000 birds collide with wind turbines per year in the U.S. [1]. There is, therefore, a need to capture the impact of wind farms on birds habitat and their migration patterns. This information can be used when deciding the appropriate locations for wind farm. In addition, accurate bird detection has a big impact on the design of collision avoidance systems in wind farms. These systems may include pulsing lights when birds are flying near the turbines [2], or lower frequencies of sound [3]. Moreover, bird counting can be improved based on the used detection method. This process is important in order to predict birds migration changes [4]. Within this context, in this paper we develop a deep learning based approach for detecting birds flying around wind turbines.

Object detection is a fundamental computer vision task, which deals with localizing, identifying and segmenting the object of interest in digital photographs. A large body of work exists that deals with this problem. The so-called traditional computer vision methods for object detection typically rely on background subtraction, thresholding, hand-crafted intensity, gradient, or shape-based features, and so forth [5]. Many of these schemes assume static backgrounds, large object sizes, or distinctive object appearances. None of these assumptions hold in the case of in-flight-bird detection. Deep learning approaches are able to *learn* features and use these to (1) localize objects, (2) predict their bounding boxes, and (3) assign object labels.

**Figure 1:**
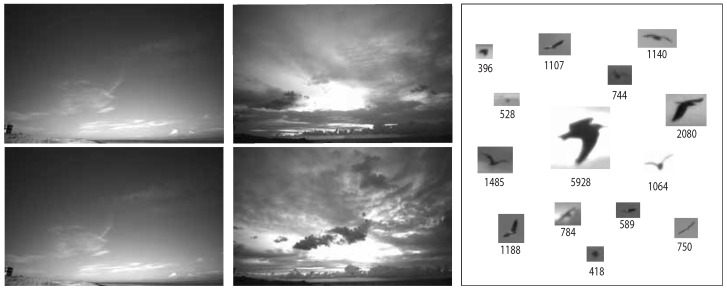
Frames from Skagen dataset (**left**). Klim dataset (**middle**) and examples of birds from both datasets and the corresponding pixels count for each box (**right**).

Region-based Convolutional Neural Network (R-CNN) inspired models have proven a wide-spread success on the tasks of objection localization, detection, and segmentation. Unlike R-CNN, which extracts detection proposals at its first stage then it refines and classifies them in a second stage, YOLO performs the two steps simultaneously [6]. Deep learning object detection methods, such as R-CNN and YOLO, needs annotated data for training and validation. In this work, we introduce two datasets collected by Mid Sweden University. The first dataset was collected at Skagen Grey Lighthouse, situated in northern Denmark, in 2018 and the second dataset was collected at Klim Fjordeholme wind farm, also in Denmark in 2017. We refer to these datasets as Skagen dataset and the Klim dataset, respectively. Figure 1 shows example frames from Skagen and Klim datasets. The difference between the two frames from each dataset is 3.4 min.

Detecting and localizing birds in flight is a challenging task. Bird flight paths span a large 3D volume and birds are often at long distances from the camera. As a result, each bird spans a small fraction of the total image pixels. Birds appearances and sizes (in pixels) also vary as they fly towards or away from the camera. Lastly, the background is not static: (1) clouds can move in and out of the field of view of a camera; (2) sky can darken or brighten due to changes in times during the day or in weather; (3) other objects, such as airplanes or drones, may enter the field of view. Figure 1 shows examples of different objects from the two collected datasets and the pixels count of each box. It is noticeable that they have different orientation and that the background changes for every object. The figure also shows the change in background and illumination, specifically in the Klim dataset.

We used Skagen dataset to train YOLO inspired models for in-flight birds detection in wind farms. The proposed deep learning model that uses temporal information for in-flight birds achieved the highest detection accuracy. The performance of our models was compared against a bird detector that relies upon background subtraction and handcrafted bird features. We show that our proposed deep learning model outperforms the background subtraction based detector.

The main goal of the study is to evaluate the suitability of classical and recent deep learning based object detection methods for the problem of birds detection in wind farms. Hence, this paper makes the following contributions. First, we introduced two new annotated datasets that can be used for developing in-flight bird detection methods. Second, we developed a background subtraction based bird detection method. We compared this method to the standard deep learning method and the proposed models for bird detection. We also used the background subtraction based method to bootstrap the data annotation process. Third, we proposed a YOLO inspired ensemble model for in-flight birds detection. Lastly, we also show that bird detection accuracy improves when the model is trained using temporal information, that is, a sequence of frames rather than a single frame. Note that our model does not assume access to tracking information between frames.

## 2. Related Works

Object detection has been studied extensively in the literature. Broadly speaking, the deep learning based object detection methods are divided into two classes: two stage [7] and single stage detectors [6]. Two stage detection detectors, such as R-CNN, Fast R-CNN, Faster R-CNN, R-FCN, and so forth, first produce region proposals. Features computed from these regions are next fed into a classifier for object detection and classification. Single stage detection detectors, such as YOLO, SSD, and RetinaNet, treat object detection as a regression problem. These are able to compute object bounding boxes and class labels in a single go. Generally speaking, single stage models have lower runtimes than two stage models. Both single stage and two stage models achieve impressive mean Average Precision (mAP) scores on different datasets. Faster R-CNN and R-FCN achieved mAP scores of 73.8% and 77.6% on the PASCAL VOC 2012, respectively [8]. Furthermore, YOLOv3 achieved mAP score of 57.9% on the MS COCO dataset. SSD has a mAP scores of 50.4% on the same dataset. YOLOv3 has better runtime performance as compared to SSD and does a better job of localizing and detecting small objects [9]. SSD is better suited to localize and detect large objects. This suggests that SSD is not the right choice for our system.

The bird collision avoidance system proposed in [10] uses stereo vision for bird detection and identification in wind farms. They used motion detection between every two consecutive frames. Then, they used a CNN that consists of two convolutional layers and two fully connected layers for class probability and identification. The birds are classified into small, medium, and large. Based on the bird category, the proposed system preforms a collision avoidance action, such activating a sound or stopping the turbine.

The authors in [11] incorporated spatial and temporal information in a spatiotemporal sampling network for object detection in videos. The network uses deformable convolutions for sampling features from adjacent (supporting) frames. The object information from two supporting frames before and after the reference frame is used to detect the object in challenging scenarios, such as blur or occlusion. Feature tensors obtained from feeding the supporting and reference frames into a deformable CNN based on ResNet-101. The tensors then are concatenated in order to predict the offset location of the object. The network was not trained using a custom dataset, but rather the benchmark Imagenet DET dataset and objects’ sizes were not stated. When testing their model, the mean Average Precision was 80.4%.

Moreover, the work in [12] addressed the issue that the majority of deep learning object detection models work with low resolution images since they are the publicly available datasets and are time efficient to train. Their goal is to detect small objects in 4k images without losing information due to downscaling. Hence, they crop the 3840×2160 frames then sending them into a YOLOv2 for initial detection. After obtaining the potential bounding boxes, active crop containing objects get fed to another YOLOv2 but with full resolution. This model was used to detect pedestrians using the PEViD dataset of UHD surveillance videos and the accuracy was 91.7%. However, it requires training two YOLO networks using different resolution, which is time consuming.

Detecting small objects is also explored in medical imaging. In order to detect skin cancer, Nie et al. in [13] compared between the different architecture of YOLOv1, YOLOv2, and YOLOv3. Their dataset consisted of 200 images of two classes, benign and malignant cancer. Their experiments show that, for that specific application, YOLOv2 achieved the heights mAP of 0.83%.

For a vehicles detection problem, YOLOv3 missed detecting small objects. In order to fix that, in [14] the authors proposed an optimized YOLOv3 network. The modified network implements a spatial pyramid pooling layer between the 5th and 6th convolutional layers to optimize its structure. Their experiments showed that testing accuracy of the model was improved by about 4% from YOLOv3.

Exploiting temporal features can improve the detection. In [15], temporal features were extracted using the difference between adjacent frames to get a full resolution saliency map of moving objects. The most salient location is selected as an attention seed and a fuzzy growing method is applied to grow the seed. Moving objects characteristic get enhanced within the saliency map and as a result the objects can be extracted. This detection method cannot be utilized for flying object detection considering that the background is dynamic; therefore, it is not possible to differentiate it from foreground objects.

Yoshihashi et al. in [16] proposed a joint framework that uses spatio-temporal information to detect and track small flying objects simultaneously. Their deep learning network consisted of four modules of convolutional, ConvLSTM, cross correlation and fully connected layers. The ConvLSTM is used to learn motion patterns from multiple frames and it improved the detection performance. The network performs region proposal based detection, and tracking of the proposals in the following frames. The implemented convolutional networks were AlexNet and VGG16. These networks are computationally very expensive.

In [17], the authors used the attention mechanism in deep learning to design a detector head. The novel dynamic head applies scale-aware attention, spatial-aware attention, and task-aware attention in order to extract scale, spatial, and task aware features. It was trained using ResNet-50 as a backbone and it can be generalized to existing one and two stage detectors. The detection accuracy of the COCO bench mark was 54.0 AP%.

It is clear from reviewing the aforementioned work that there is as a need to investigate and improve methods for detecting small flying objects in large volumes. This work presents a robust convolutional neural network (CNN) based method for detecting flying objects in order to enable sky surveillance.

## 3. Detection Evaluation

A commonly used metric for object localization and detection is mean Average Precision (mAP) [18]. Specifically, the Pascal VOC 2010–2012 [19] which samples a curve at all precision and recall values. The mAP is then calculated as the exact area under the precision-recall curve (AUC). To determine whether a prediction is correct or not, Intersection over Union (IoU) is used. IoU is the ratio of (1) the overlap in pixels of the predicted bounding box and the ground truth bounding box to (2) the union in pixels of the predicted bounding box and the ground truth bounding box.

YOLO uses the objectness (confidence) score concept. It is the network’s confidence that an object exists in the given box. A prediction is True Positive (TP) if: its objectness score is greater than or equal to some confidence threshold, the predicted class matches the class of the ground truth, the IoU with ground truth is greater than or equal to the IoU threshold. A prediction is False Positive (FP) if either of the latter two conditions is not true. Precision is the percentage of TF among all predictions, and recall is the percentage of TF among the ground truths. The mAP metric in the Pascal VOC 2010–2012 interpolates all the points to calculate the AUC of the precision-recall curve. By default, the IoU threshold in this calculation is 0.5. Mathematically,

$$\begin{array}{cc} Precision & =\frac{TP}{TP+FP}\\ Recall & =\frac{TP}{TP+FN} \end{array}$$

where FP and FN are false positives and false negatives, respectively.

Another evaluation metric that is used by the MS COCO challenge is Average Precising (AP) [20]. In this method, only 101 recall points are interpolated for each class at different IoU thresholds. We mainly used mAP from the VOC 2010–2012 for accuracy evaluation. Also, we reported the AP from the MS COCO challenge. Both metrics are tested at an IoU threshold of 0.5. For our unique datasets of small objects in 4K frames, the amount of 50% intersection is enough to detect small birds. For example, having a 0.75 IoU in AP_75_ would cause the models to miss detecting small objects.

## 4. Materials and Methods

We developed three YOLOv4 based models for bird detection, and we compared the performance of these models against the background subtraction based object detection method described in Section 4.2. In the subsequent discussion, we refer to these YOLOv4 based models as Model 1, Model 2, and Model 3.

### 4.1. Dataset Acquisition and Camera Setup

Deep learning object detection methods need annotated datasets for training, validation, and testing. We did not find any dataset that can be used to study in-flight bird detection methods presented in this paper. Consequently, we decided to collect our own dataset. The datasets used in this work were collected at two sites in Denmark over the course of a few months in 2017 and 2018. For the data acquisition process in this work, we used the setup in our previous work [21]. We manually annotated the dataset using the freely available *LabelImg* online tool [22].

**Figure 2:**
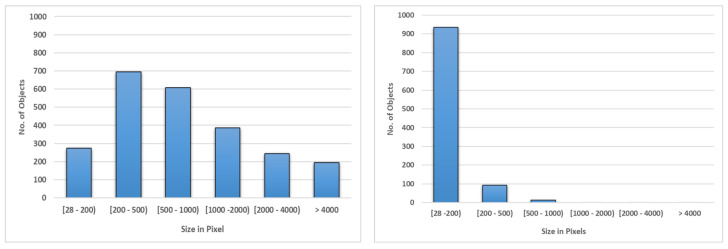
Bird size statistics from Klim (**left**) and Skagen (**right**) datasets.

The Skagen dataset was collected at the *Skagen Grey Lighthouse, Center of Migratory Birds* using a pair of wide-angle, monochrome cameras firmly affixed to rigid boxes. The cameras were connected to Nvidia Jetson TX2 edge-computing device recording 4K images at 5 fps. The cameras were facing upwards. This dataset exhibits a relatively stationary background in terms of cloud movement and illumination changes, Figure 1 (left). The Kilm data were collected at *Klim Fjordeholme* using the same camera setup; except, here, the cameras are mounted on tripods. The dataset exhibits a more dynamic background in terms of cloud movement and variations in illumination, as shown in Figure 1 (middle). Birds seen in these datasets have smaller sizes than objects in widely used object detection datasets, such as MS COCO and Pascal VOC [23]. For example, in the Kilm dataset 1000 out of 2400 bird bounding boxes have areas less than 500 pixels (see Figure 2).

While both datasets contain hours of video footage, it was infeasible to use all of this data for training or testing. We decided to use 6 min video from the Klim dataset to develop and test the proposed models. The video was comprised of 1700 frames with 2400 birds. Figure 2 plots a histogram of bird bounding box sizes in pixels from both dataset. It is clear that bird sizes in Skagen are smaller than those in the Klim dataset. Majority of them are within 200 pixels. We used 5 min of video from Skagen dataset to test over proposed methods. We did not use frames from Skagen dataset for training.

### 4.2. Background Subtraction for In-Flight Bird Detection in Monochrome Videos

The method comprises four steps: (1) background subtraction, (2) camera gain compensation, (3) noise removal, and (4) blob detection. Background subtraction module maintains a background model and uses it to identify the foreground regions in the current image [24]. OpenCV morphological gradient operation is applied to the foreground regions as a post-process to compensate for pixel intensity changes due to camera gain or exposure parameters [25]. Morphological gradient is the difference between dilation and erosion of the frame. The resulting frame is then blurred to smooth it and reduce the noise. For that we used Gaussian blur with kernel of size 9. OpenCV blob detector operation estimates the centers and radii of the foreground regions. A major shortcoming of this approach is that foreground regions with areas between a certain user-defined range are tagged as birds. The method is outlined in Algorithm 1.
**Algorithm 1.** Using background subtraction for bird detection and localization in monochrome video frames.1: B←(Background model)
2: I←(Current frame)
3: D←I−B
4: $D_{morphology}\leftarrow \mathrm{Morph}\left(D\right)$
5: $D_{blur}\leftarrow \mathrm{Blur}\left(D_{morphology}\right)$
6: $M\leftarrow \mathrm{DetectBlob}(D_{blur})$
7: List of bounding boxes←Filter(M)



This method does not use size, shape, or color information for bird detection. Bird sizes vary as the distance between the bird and the camera changes. Bird shapes and orientations also change. Furthermore, shapes are not easily discernible, since most birds span a tiny fraction of the total image pixels. We also do not have access to color information, since our cameras capture monochrome video.

### 4.3. Model 1: YOLOv4

In this work, we chose YOLOv4, which is the most recent iteration of YOLO architectures at the time of this study [26]. YOLOv4 is comprised of a backbone network, a neck network, and a head network. It uses CSPDarknet-53 as the backbone network. Darknet-53 is 53 convolutional layers deep and uses Spatial Pyramid Pooling (SPP) for feature extraction [27]. Path Aggregation Network (PANet) serves as the neck network [28]. It collects features from different layers of the backbone network. YOLOv3 is used as the head network for YOLOv4.

**Figure 3:**
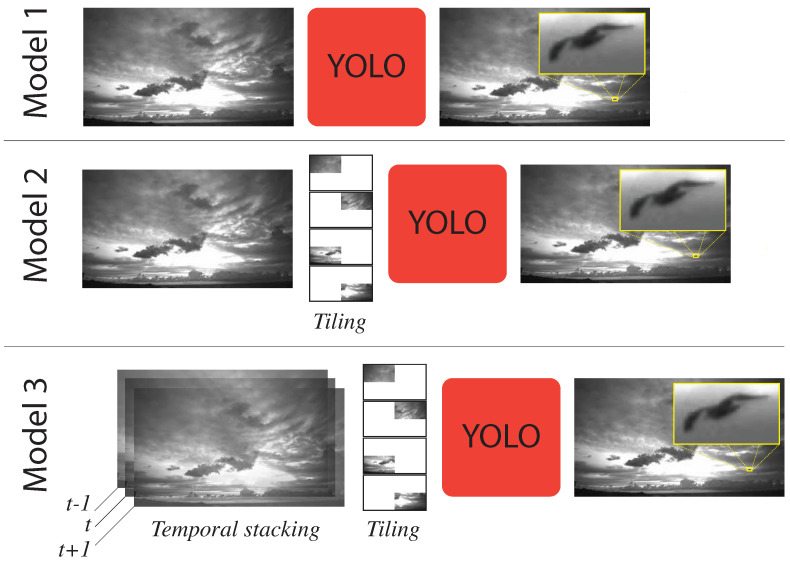
Three YOLOv4 based models, each trained on the Klim dataset, for bird detection around wind farms.

Model 1 shown in Figure 3 (top-row) takes the full 4K frame as input. The input frame is resized to 1024×1024 image and passed to YOLOv4 model, which is trained on the Klim dataset. The original YOLOv4 is trained using the COCO dataset [20]. Unlike COCO, the Klim dataset consists of a single class label. Majority of the objects in this dataset are small objects as shown in Figure 2, model 1 performed poorly detecting small birds (i.e., birds that appear small in the image). This can perhaps be explained by the fact that information about these birds is lost when images are resized from 4K 3840×2160 to the network resolution of 1024×1024. This downsampling reduces the size of smaller birds to less than the size of the smallest anchor boxes. We address this issue in Model 2 described below.

### 4.4. Model 2: Tiling + YOLOv4

Model 2 depicted in Figure 3 (middle-row) splits the 4K input image into four 1920×1080 images. Each of these images is then resized to the network resolution of 1024×1024 and is fed into YOLOv4 pipeline. The detection results of the four tiles is combined to form the final output. This model was also trained on the Klim dataset. The results presented in the following section suggests that this model does a better job in detecting small birds as compared to Model 1. We currently assume that birds at the tiling boundaries are a rare occurrence, also that the birds do not stay long at these boundaries. The model, therefore, does not handle the situation when a bird strides one of the four tiling boundaries. We leave this as future work.

### 4.5. Model 3: Temporal Information + YOLOv4

Neither Model 1 nor Model 2 exploit the fact that we are in fact dealing with a video. Both models treat each frame independently. As a result these models do not take advantage of temporal information across frames. This temporal information provides a power cue to deal with issues due to motion blur, camera defocus, and changes in sizes [6]. Model 3 combines information from frames at times t−1, *t*, and t+1 and constructs a w×h×3 input frame that is fed into YOLOv4. Model 3 also uses tiling similar to Model 2 above. Therefore, given 3 4K images, four 1920×1080×3 images are constructed. Each image is then resized to 1024×1024×3 and passed into YOLOv4 pipeline. Results from the YOLOv4 pipeline are combined to create the final output. Model 3 is shown in Figure 3 (bottom-row).

### 4.6. Model Training

We employed *transfer learning* to train our models. Specifically, we started with YOLO weights trained on the MS COCO dataset. We used the YOLOv4 pre-trained weights file (yolov4.conv.137) which freezes the wights up to convolutional layer number 137, one layer before the first YOLO layer, and train the rest of the layers using our custom dataset. YOLOv4 performance depends upon a number of hyper parameters. We performed a grid search to find optimal values for the following parameters: batch size, subdivision, network resolution, learning rates, and anchors.

The models were implemented on Google Colab Pro cloud services, alternating between Tesla P100-PCIE and V100-SXM2 16GB GPUs. The hyperparameter were set as follows: batch size: 64, sub-division: 32, height and width: 1024, momentum: 0.949, decay: 0.0005, learning rate: 0.001, max batch: 2000. As we have only one class, the number of filters before each of the three YOLO layers is set to 18.

For a custom object detector, anchors are important parameters to tweak based on the object sizes in the annotated training dataset. In YOLO, anchor boxes are estimated using k-means clustering with cluster size of 9 on the dimensions of the ground truth bounding boxes. Each model has 9 anchor boxes to learn small, medium, and large objects. The optimized parameters for each model are listed in Table 1.

Table 2 presents the datasets used to train, validate, and test each of the three models, in terms of number of frames and number of ground truth bounding boxes. The difference in frames and boxes number among the models is due to tiling in Model 2 and temporal features calculation in Model 3. Tiling results in a large number of empty frames. We did not include all of these empty frames in the training. For a given frame in Model 3, we used the information from the previous and next frame. So the first and last frames cannot be included. Also, most of the frames have multiple objects (boxes). We divide the annotated dataset for each model as follows: 80% for training, 10% for validation, and 10% for testing.

Moreover, k-fold cross validation [29], is used to make sure that Model 3 is not overfitting and that it can be generalized to new datasets within the problem domain. In order to optimize the model inference, we perform a models ensemble for the results from cross validation.

### 4.7. Ensemble Models

To further reduce overfitting and improve detection performance, we set up an ensemble from the *k* fold cross validation of Model 3. Our ensemble model comprised of six models, each trained using a different subset of the training data. We used an open-source library that ensembles detection at inference [30]. A number of detections are predicted for the same object from the six folds. A voting strategy is then used to decide whether the prediction is an object. The three strategies are *Affirmative*, which is when any of the six models detect the object. *Consensus* is when more than half of the models agree upon a detection. Last, *unanimous* is when all the six models detect the object.

## 5. Results

**Figure 4:**
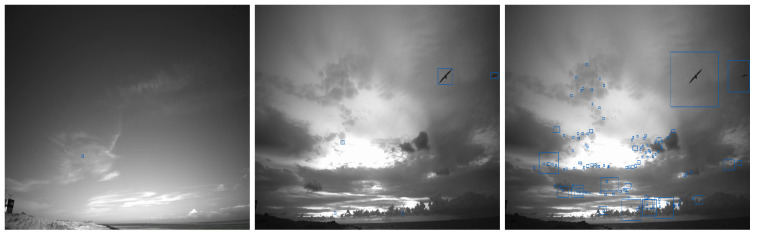
Background subtraction based model for bird detection. Bird detection on a frame from Skagen dataset (**left**). Ground truth bird bounding boxes on a frame from the Klim dataset (**middle**) and detection results exhibiting a large number of false positives (**right**).

Background subtraction based model achieved precision and recall values of 84% and 60% on Skagen dataset. This model, however, performed very badly on the Klim dataset. Skagen dataset exhibits a relatively static background, whereas Klim dataset exhibits highly dynamic background due to cloud movement and changes in illumination, as shown in Figure 1. This suggests that background subtraction based bird detection model is unable to deal with settings where birds are viewed against a dynamic background. Figure 4 (right) highlights the failure of the background subtraction based model on a frame from the Klim dataset with a large number of false positives. Note that this model does well on the frame from Skagen dataset, Figure 4 (left).

Model 1 is trained using 4K frames from the Klim dataset. The dataset includes 1607 objects for training, 340 for validation, and another 357 for testing. This model achieved a mAP score of 83.7% on the validation set. The default objectness score (confidence score) that all predicted boxes are thresholded by is 0.25.

Objectness score is the network’s confidence that an object exists in the given box.

The testing mAP score is 82.9% on the unseen data. Model 2 splits the 4K images into four 1920×1080. These images are subsequently resized to 1024×1024 and fed into YOLOv4. For Model 2, we used 1120 objects for training, 260 for validation, and 231 for testing. This model achieved a mAP score of 88.7% on the testing data. Model 3 used temporal information stacking across frames in order to improve bird detection accuracy. Model 3 achieved mAP score of 90% on the test data. This is 2% improvement over Model 2 and 8% improvement over Model 1. Results for Model 1, 2 and 3 are summarized in Table 3. The table also shows the accuracy using COCO’s AP_50_, which is very close to the mAP results of VOC 2010–2012 for all models.

We used Model 3 trained on six subsets of the Klim dataset to set up an ensemble model. Specifically, we divided 1200 frames from the Klim dataset into six groups. Data in each group is used to train a Model 3. The six trained models form an ensemble model. The ensemble model was then evaluated on a test dataset. We used (1) Consensus, (2) Affirmative, and (3) Unanimous schemes to construct the final outcome of this ensemble model [30]. In Consensus, more than half of the models need to agree on the outcome (i.e., a bird detection). In Affirmative, bird detection from a single model is sufficient for the ensemble to output a bird detection. In Unanimous, all models must output a bird detection for the ensemble to output a bird detection. The mAP values for the ensemble model on the Klim dataset are summarized in Table 4. Affirmative scheme for constructing the final outcome achieves the highest mAP value of 94%. This is interesting and it seems to suggest that false negatives are more common than true negatives in our models. This merits further inquiry. Results shown in Table 5 confirm that the ensemble model outperforms each of the six constituent models. Here, K*x* refers to Model 3 trained on partition *x* of data, where x∈[1,6].

Finally, Table 6 summarizes testing mAP values for background subtraction based method, Model 1 and Model 3 from both Skagen and Klim datasets. Note that neither Model 1 nor Model 3 were trained on the Skagen dataset. Background subtraction based bird detection method achieved mAP value of 71% on Skagen dataset; however, this method did not do well on the Klim dataset. Model 1 achieved mAP value of 60% on Skagen and 82% on Klim dataset. This model was not trained on the Skagen dataset. Model 3 performed the best out of the three. This model achieved mAP values of 92% and 94% on Skagen and Klim datasets. This model was never trained on the Skagen dataset and yet achieved a very good testing result of 92%.

## 6. Discussion

**Figure 5:**
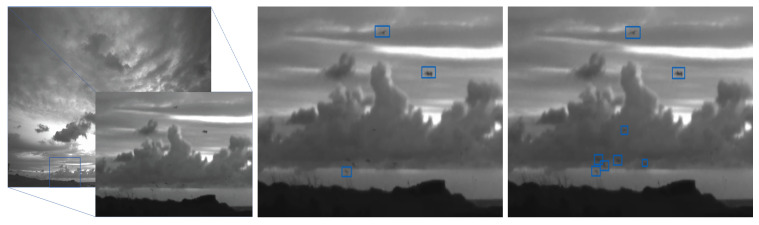
The effect of tiling on accuracy. Cropped region from the input frame with ground truth (**left**). Model 1 detection results cropped to match the input frame (**middle**) and Model 2 prediction results (**right**).

*Tiling* helps the YOLOv4 based bird detector find small birds. When tiling, the 4K input frame is split into four 1920×1080 frames. Each of which is passed independently to the YOLOv4 based detector. We refer to this setup as Model 2. Figure 5 highlights the benefits of tiling. Here, the input frame with a zoomed in part containing ground truth objects is shown on the left. The middle frame shows the detection results when the model is trained without tiling (i.e., Model 1), and the right frame shows the detection results when model is trained with tiling. Recall that the input size for each YOLOv4 based bird detector is 1024×1024, and it seems that resizing the full 4K image to 1024×1024 diminishes the salient aspects of small birds.

**Figure 6:**
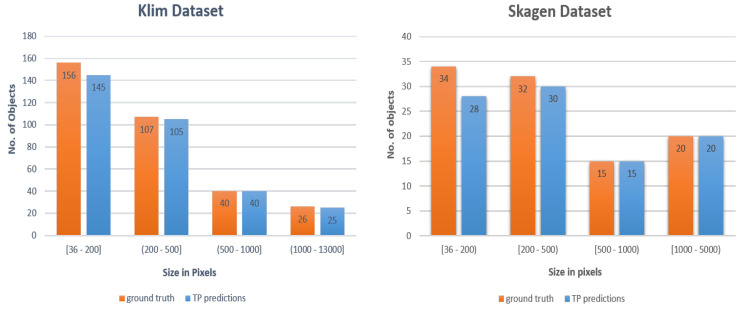
Size and number of ground truth and true detected objects using Model 3 for Klim (**left**) and Skagen (**right**) testing datasets.

*Temporal stacking* also helps improve bird detection. Model 3 uses both tiling and temporal stacking where frames at t−1, *t*, and t+1 are stacked on top of each other and fed into a YOLOv4 based bird detector. Model 3 (and its ensemble versions) achieves better mAP values as compared to those achieved by the other three approaches: background subtraction based method, Model 1 and Model 2. For the Klim test dataset of 330 birds, Model 3 was able to correctly detect 315 birds. Furthermore, for 156 birds between the sizes of 36 to 200 pixels, 145 were correctly detected. In other words, Model 3 was able to correctly detect 93% of the birds in the smallest category on the Klim test dataset (Figure 6 right). In addition, Model 3 only missed eight birds in the Skagen test dataset (Figure 6 left).

### Background Subtraction vs. YOLOv4 for Bird Detection

**Figure 7:**
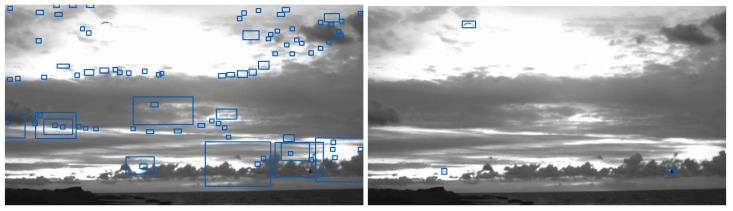
The effect of background and illumination variant on detection using computer vision based detector (**left**) and deep learning Model 3 (**right**).

We also conclude that background subtraction based bird detection method is unable to deal with changes in background. Specifically, this method generates a lot of false positives. The YOLOv4 based method, and in particular Model 3 and its ensemble variant, is able to deal with dynamic backgrounds. Figure 7 illustrates this observation. Note that the background subtraction based method (left) detects many false positives for the same frame. In reality there are only three birds present in the image and all of them were correctly detected using Model 3 as in Figure 7 (right).

## 7. Conclusions

This paper tackles the problem of bird detection in grayscale videos captured by cameras mounted around wind turbines. Specifically, we presented a background subtraction based bird detection method, plus three increasingly capable YOLOv4 inspired models. We evaluated these models on Klim and Skagen datasets, also introduced in this paper. It is worth noting that the Skagen dataset was only used for testing purposes. Our results suggest that both *tiling* and *temporal stacking* improve bird detection performance. Model 3 that uses both tiling and temporal stacking achieves the highest mAP values of all methods on both datasets. We also proposed an ensemble model that comprises six Model 3 trained on different segments of the Klim training set. The ensemble model further improves the mAP value achieved by Model 3. Our tiling strategy currently cannot deal with situations where a bird straddles the tiling boundary. In the future, we plan to deal with this issue. The proposed model can be implemented in surveillance systems to detect small flying objects, such as in wind farms.

## Figures and Tables

**Table 1:** Training parameters for Model 1, 2 and 3.

	Model 1	Model 2	Model 3
burn in	100	400	400
steps	(1600, 1800)	(1400, 1700)	(1400, 1700)
scales	(0.1, 0.1)	(0.1, 0.01)	(0.1, 0.01)
anchors	(4,5) (6,7) (9,9) (8,14) (14,12) (15,20) (24,25) (32,46) (74,121)	(5,8) (7,11) (11,13) (13,18) (20,18) (20,29) (33,27) (50,47) (60,98)	(5,8) (7,11) (11,14) (15,17) (21,20) (19,31) (32,27) (48,46) (61,97)

**Table 2:** Klim dataset. Training, validation, and test splits for models 1, 2 and 3. Both validation and testing comprise 10% each of the total dataset.

	Model 1			Model 2			Model 3		
	Training	Validation	Testing	Training	Validation	Testing	Training	Validation	Testing
Frames	1280	150	170	1068	133	133	1060	125	125
Boxes	1607	340	357	1120	266	231	1108	258	223

**Table 3:** AP_50_ and mAP scores for Model 1, 2 and 3 on the Klim dataset. Here M1, M2, and M3 refer to Models 1, 2 and 3, respectively.

	Training/Validation/Test Set	${AP}_{50}$ (%) (Validation)	mAP(%) (Validation)	${AP}_{50}$ (%) (Test)	mAP(%) (Test)
M1	1607/340/357	83.6	83.7	82.4	82.9
M2	1120/266/231	82.5	82.8	88.4	88.7
M3	1108/258/223	77.9	78.1	89.6	**90.1**

**Table 4:** mAP scores of the ensemble model on the Klim dataset using three different methods for constructing ensemble outcome.

	Voting Strategy		
	**Consensus**	**Affirmative**	**Unanimous**
mAP (%)	93	**94**	85

**Table 5:** mAP values for Model 3 trained one of six partitions of the Klim dataset. These six models form the ensemble model.

	Training/Validation/Test Set	mAP (%) (Validation)	mAP (%) (Test)
K1	1188/198/330	90.7	92
K2	1258/128/330	95.9	89.4
K3	1285/101/330	85.6	88.6
K4	1262/124/330	88.8	84.4
K5	1028/358/330	78.3	86.4
K6	909/477/330	85.7	87.5

**Table 6:** Accuracy of the testing set using computer vision and deep learning.

	mAP (%) (Skagen)	mAP (%) (Klim)
Background subtraction	71%	<1%
Model 1	60	82
Model 3	92	94

## Data Availability

The used datasets will be available at: https://github.com/STCResearchCentre/datasets, (accessed on 26 October 2021).
